# Non-encapsulated, encapsulated, and lyophilized probiotic *Limosilactobacillus reuteri* SW23 influenced the growth and gut health in calves

**DOI:** 10.1038/s41598-024-57353-y

**Published:** 2024-04-01

**Authors:** Manish Yadav, Sachin Kumar, Yash Parsana, Nutan Chauhan, Nitin Tyagi, Goutam Mondal, Ashis Kumar Samanta

**Affiliations:** Division of Animal Nutrition, ICAR-National Dairy Research Institute, Karnal, Haryana 132001 India

**Keywords:** Microbiology, Molecular biology, Animal biotechnology

## Abstract

The present study was conducted to assess the impact of non-encapsulated, air-dried microencapsulated, and lyophilized microencapsulated probiotics in indigenous cattle calves (*Bos indicus*). Twenty-four (5–7 days old) indigenous cattle calves were selected and assigned into four groups, with six calves in each as follows: control (CON), fed milk and basal diet alone, and treatment groups supplemented with non-encapsulated (NEC), air-dried microencapsulated (AEC) and lyophilized microencapsulated (LEC) probiotic *L. reuteri* SW23 at 10^8^ CFU/head/day in skim milk as a carrier provided for 60 days. The animals were divided into four groups, adopting a complete randomized design, and the effects were considered significant at *p* ≤ 0.05. Probiotics supplementation increased (*p* < 0.05) body weight gain (kg), average daily gain, and structural growth measurements in calves of all treatment groups. Dry matter intake (g/d), feed conversion efficiency, and fecal counts of *Lactobacilli* and *Bifidobacteria* were also increased in the treatment groups compared to CON. The fecal consistency index was highest in CON (0.70 ± 0.03), followed by NEC (0.68 ± 0.01), AEC (0.66 ± 0.02), and LEC (0.65 ± 0.02). Fecal pH and ammonia levels were reduced (*p* < 0.05) in the probiotic-fed groups compared to CON, with a concomitant increase in fecal lactate, acetate, and propionate levels. In addition, cell-mediated and humoral immunity were significantly increased in supplemented groups as compared to CON. Thus, it can be concluded that supplementation of the probiotics in microencapsulated/non-encapsulated forms to neonatal calves had a variety of positive effects on their health, including better performance, improved gut health, and a lower fecal consistency index. Moreover, among all supplemented groups, the lyophilized microencapsulated group outperformed air-dried microencapsulated and non-microencapsulated groups in terms of ADG, DMI, and gut health.

## Introduction

Calf rearing is the most crucial activity of the livestock unit because calves are the future production entity coupled with major income generating platform of farms. Thus, it is essential to employ suitable nutritional interventions to foster the calves' growth and well-being^1^. The digestive system of newborn calves receives microbiota from the birth canal of the dam and the surrounding, which populates the gastrointestinal tract (GIT) of newborns^2^. Neonatal calves are very sensitive during early life, particularly to sudden changes in the environment or nutrition, or other types of managemental stress; causing aberration of gut microbiota^3,4^. Microbial imbalances may promote the growth of pathogenic and opportunistic bacteria as well as the emergence of several illnesses with harmful effects on the host^5,6^. A major issue for sustainable cattle production is calf mortality. Out of the 5% overall death rate for pre-weaned calves, digestive issues, and scours account for about 56%, while respiratory issues and others account for the remaining 44%^7,8^. Since forties of previous century, the use of antibiotics at sub-therapeutic and therapeutic dosages has been a frequently used strategy for dealing with pathogenic bacteria concerns in farm animals. However, growing antimicrobial resistance (AMR) concerns around the world have compelled researchers of animal sciences and relevant stakeholders to look for other possible substitutes^9,10^. Additionally, the EU has banned the use of antibiotics as feed additives as of January 1, 2006 (Commission Regulation EC No 2200/2001), which has increased the need for antibiotic substitutes.

To address these issues, probiotics have been proven to be a beneficial means of enhancing intestinal health, and general well-being, increasing digestive efficacy, increasing growth performance, and maintaining overall health^11,12^. Numerous studies conducted previously in authors’ laboratory also demonstrated that feeding probiotics to pre-ruminant calves improves both their growth performance, gut health, and immune response^1,13–16^. A good immune response depends on the harmony between humoral and cell-mediated immunity, and probiotics can stimulate both of them^9^. Recently, autochthonous or host-specific probiotics have received more attention than their allochthonous probiotics^17–21^ because the former colonize the host intestine more readily due to their evolutionary adaptation to the ecological niche of the host gut^22,23^.

For optimal colonization and proliferation, orally delivered probiotics must reach the host target region at a concentration of at least 10^6^–10^7^ colony-forming units (CFU)/g^24^. The probiotic content in probiotic-enriched functional feeds must therefore be in the range of 10^8^–10^9^ CFU/g at the time of intake^25^. Due to the hostile GIT environment, which includes an acidic pH, bile salt, etc., live probiotic cultures may undergo a loss in CFU by the time they reach the gut and may be unable to fully display their biological activity^26^. The scientific community and industry have been looking for options to protect probiotic microorganisms to prevent loss of vitality during processing, storage, and digestion^27^. One such popular method is the microencapsulation of probiotic live cells. This technique can shield the live bacteria by coating them with appropriate materials such as sodium alginate, carrageenan, pectin, starch, chitosan, gum, etc., to maintain their viability and effectiveness during their manufacturing, storing, and digestion processes and allow them to exert their probiotic effect in the gut at an adequate dose level^28^. Spray-drying and lyophilization (freeze-drying) are typically the most widely used technologies; however, other techniques like extrusion, emulsion, and spray-chilling are also effective^28^. Because the heating process produces a high temperature, using that technique on probiotics is inappropriate through the spray-drying chamber as probiotics' are extremely sensitive to heat^29^. To effectively dehydrate the samples, while minimizing heat stress on the bacteria, lyophilization is a method that can sublimate the water content in the chilled samples to a gaseous state under vacuum circumstances^30^.

*Lactobacillus* and *Bifidobacterium* are the most commonly used probiotics. *L. reuteri* is a Gram-positive, lactic acid-producing, hetero-fermentative species that lives in the GIT of animals and is considered as one of the few autochthonous (native) species of *Lactobacillus* in animals that can be utilized as probiotics^13^. *L. reuteri* SW23 (NCBI GenBank accession number—MW074915), a previously identified promising probiotic strain^18,^ was selected based on probiotic attributes. In this context, recently, an autochthonous probiotic *L. reuteri* SW23 was microencapsulated in the chitosan-coated alginate-inulin matrix in the author’s laboratory and characterized in vitro by Parsana et al.^25^. Evidently, the particular findings suggested for additional research to determine the usefulness of microencapsulated probiotics in vivo models. On the other hand, little is known regarding the efficacy of the autochthonous probiotic forms, including non-encapsulated, microencapsulated air-dried, and microencapsulated freeze-dried (lyophilized) in calves. It is hypothesized that different forms of probiotics, namely non-encapsulated, encapsulated, and lyophilized, will have varying effects on the gut health and productivity of calves. Hence, the current study was planned to investigate the effects of various processed forms of the autochthonous probiotic *L. reuteri* SW23 on growth and gut health in calves.

## Materials and methods

### Ethical approval

All procedures performed in studies involving animals were in accordance with the ethical standards of the Institutional Animal Ethics Committee (IAEC) of ICAR-National Dairy Research Institute, Karnal, Haryana, India, under Reg No. 1705/GO/ac/13/CPCSEA dated 03/07/13. The authors confirm that the study is reported in accordance with ARRIVE guidelines. The experimental protocol was approved and conducted as per the guidelines laid down by Institutional Animal Ethics Committee of ICAR-National Dairy Research Institute, Karnal, Haryana, India.

### Selection, revival, microencapsulation, and lyophilization of probiotic strain

*Limosilactobacillus reuteri* SW23 (NCBI GenBank accession number—MW074915), a previously identified promising probiotic strain^18,^ was selected, and its purity was examined. 0.1 mL of *L. reuteri* SW23 from stock cultures were added to 10 mL of de Man, Rogosa, and Sharpe (MRS) broth, and the mixture was then incubated anaerobically at 37 °C for 24 h. The strain was three times subcultured, inoculated (1 mL) in 100 mL of MRS broth, and incubated anaerobically for 24 h at 37 °C. To obtain a cell concentration of 10^10^ CFU/mL, the cells were collected, washed twice in sterile PBS solution, and then centrifuged for 20 min at 4 °C at 11,31 × g. The probiotic was microencapsulated using the extrusion process in an alginate-inulin matrix and subsequently coated with a chitosan layer, as explained by Parsana et al.^25^. In brief, after washing, *L. reuteri* SW23 cell suspension was combined with 5 mL of sterile 2% inulin (MP Biomedicals, Cat No:198971; Purity:92.8%) solution and 20 mL of sterile 3% sodium alginate solution. Then, a 24 G needle was used to extrude this combination dropwise into a sterile 0.1 M calcium chloride solution under stirring (Magnetic stirrer, Scilogex, MS-H-Pro, Genetix Biotech), at 1×g. The resultant microcapsules were allowed to rest for 30 min, filtered using Whatman filter paper no. 4, and then rinsed with sterile PBS solution to get rid of any calcium chloride that was not already reacting. The microcapsules were then given a second coating with chitosan. Freshly manufactured microcapsules were air-dried by exposing them to laminar airflow for overnight night to prepare air-dried microencapsulate or probiotic microcapsules that were lyophilized as described by previous researchers^31,32^ for feeding to the animals, as detailed in the next section.

### Animal, diet management, and experimental design

The current animal experiment of sixty days duration was carried out at the Livestock Research Centre of the ICAR-National Dairy Research Institute, Karnal, Haryana, India. The optimum requirements in terms of temperature (18–21 °C), air changes (8–12 per h), lighting (12 h light/dark cycle), and humidity (35–70%) were maintained in the calf pen for comfortable conditions in the housing as per the farm practices. Briefly, the neonatal calves were separated from their mothers following colostrum feeding and housed in individual calf pens (1.6 × 2.5 m) having a layer of wood shavings to create a comfortable environment. Cleaning of the calf pens occurred twice daily, and disinfection using a diluted phenyl solution was conducted twice a week. Before commencing the research experiment, a general health assessment (body temperature, body weight, normal behaviour, normal respiration) in the presence of a trained veterinarian was carried out on each calf to identify any signs of illness or injury. The study specifically enrolled only those calves that were deemed healthy for the experiment.

Twenty-four cattle calves (5–7 days old with an average BW of 25 ± 1.0 kg) of different indigenous breeds (Sahiwal, Gir, and Tharparkar in equal numbers) were chosen and randomly allocated into four groups of six animals each, with an equal ratio of male and female calves and an equal ratio of different breed. The calves received the following supplements along with the basal diet: Group I (CON) received no supplementation; Group II (NEC) received non-encapsulated probiotic (1 mL/calf/day with 10^8^ CFU/g); Group III (AEC) received air-dried microencapsulated probiotic (1 g/calf/day with 10^8^ CFU/g) and Group IV (LEC) received freeze-dried (lyophilized) microencapsulated probiotic (1 g/calf/day with 10^8^ CFU/g). All forms of probiotics were given to the treated animals orally before their morning meal for 60 days after being diluted with skim milk as a carrier.

Quality dietary ingredients comprising maize, mustard oil cake, groundnut, soybean meal, bajra, rice polish, wheat bran, salt, vitamin, and mineral premix (Table 1) were used in the formulation of the calf starter (concentrate mixture), which was offered to the animals from the second week onwards on a stainless steel feeding trough. Throughout the length of the trial, all of the calves received ad libitum calf starter and freshly harvested chafed green fodder (maize/sorghum) following the feeding protocol suggested by Sharma et al.^1^ and Singh et al.^33^. All the animals also had unrestricted access to clean drinking water. Each animal was housed in an individual pen. To keep it dry, the pens were cleaned twice a week with a diluted phenyl solution, and manure was collected twice daily.

**Table 1:** Chemical composition (on % DM basis) of the basal diet and milk fed to calves.

Nutrients	Calf starter (concentrate mixture)	Green fodder	
		Maize	Sorghum
DM	89.40	24.50	27.20
OM	91.40	90.40	93.50
CP	22.70	9.80	8.60
EE	4.40	2.30	1.60
NDF	24.20	63.10	61.30
ADF	14.30	30.40	32.40

Ingredients proportions (%): 28 maize, 5 bajra, 10 groundnut, 15 soybean meal, 13 mustard oil cake, 15 wheat bran, 11 rice polish, 2 vitamin and mineral premix, 1 salt.

Premix provided per kilogram of concentrate: vitamin A, 15,000 IU; vitamin D, 5000 IU; vitamin E, 50 mg; Fe, 90 mg; Cu, 12.5 mg; Mn, 30 mg; Zn, 90 mg; Se, 0.3 mg; I, 1.0 mg.

DM, dry matter; OM, organic matter: CP, crude protein; EE, ether extract; NDF, nutrient detergent fibre; ADF, acid detergent fibre.

### Growth performance and nutrient utilization

A digital electronic weighing balance was used to measure the animals' body weight (BW) weekly before morning feeding. The net gain in BW and average daily gain (g/d) were calculated. The body length, heart girth, withers height, and hip height of each calf were taken weekly using “tape measure”^1^. Daily measurements of feed intake were performed, and after determining the dry matter (DM) content of the offered feed and the residue left behind, the mean dry matter intake (DMI) of each calf was computed. To ascertain the digestibility of the nutrients, a digestion trial comprising a 5-d collecting period was conducted from days 55 to 59 toward the end of the feeding experiment. For each calf, 24-h DMI was registered and feces were collected directly from the floor separately in plastic containers, and the total amount of feces per day was recorded. Using the formula (nutrient intake − nutrient output/nutrient intake × 100), the apparent digestibility of various principles viz. dry matter; organic matter; crude protein; ether extract; neutral detergent fibre; and acid detergent fibre was calculated. According to the Association of Official Analytical Chemists' standard operating protocols, proximate principles of feed and feces were analyzed^34^.

### Fecal characteristics

Calves were assessed daily for fecal consistency using a scale ranging from 1 to 4 as described by Meyer et al.^35^, where 1 is "normal" meaning the feces are firm but not hard and slightly deformed when falling and setting on the floor; 2-soft: shapeless feces that pile up and then partly scatter after falling; 3-liquid: feces that spread out in sheets 6 mm deep and 4 is watery feces with a liquid consistency (with diarrhea). The fecal consistency index was calculated based on the following formula.

$$FCI=[\left(dE1\times 1\right)+\left(dE2\times 2\right)+\left(dE3\times 3\right)+\left(dE4\times 4\right)/Td\times 4$$

where Td is the total number of days in the experiment (Td = 60), and dE1, dE2, dE3, and dE4 are the number of days with fecal consistency scoring of 1, 2, 3, and 4, respectively.

Fecal samples were collected at fortnightly intervals^36^ and the pH of feces samples was directly determined using a digital pH meter (Model: pH Spear, Eutech Instruments, Malaysia) designed exclusively for detecting the pH of semi-solid substances. The fermentative ends products such as fecal short-chain fatty acids (SCFAs), lactate, and ammonia were performed by preparing different aliquots as per the methodology given by Kore et al.^37^. In brief, 6 mL of 6.0 N HCl solutions were added to 2.0 g of fresh feces and stored at − 20 °C to perform ammonia estimation later following the protocol devised by Chaney and Marbach^38^. For the examination of fecal short-chain fatty acids (SCFAs), 2 g of fresh feces were combined with 4 mL of 25% (w/v) metaphosphoric acid, which was then centrifuged for 10 min at 5590×*g* and the produced supernatant was kept at − 20 °C for further analysis following the method of Cottyn and Boucque^39^. Another, 2 g of fresh feces was diluted with 4 mL of distilled water and centrifuged at 5590×*g* for 10 min, and the supernatant was stored at − 20 °C for lactate analysis^40^.

### Fecal microbiota

The counting of fecal select bacterial populations was performed by vortexing 1 g of homogenized fresh feces in 9 mL of normal saline (0.9% NaCl solution). Bacterial populations were counted using the serial dilution method (10^1^ to 10^8^) in duplicate by plating in the following media: reinforced clostridial agar (Himedia, Mumbai, India) for clostridia, deMan, Rogosa, and Sharpe agar (Himedia) for lactobacilli^41^, MacConkey agar (Himedia) for coliforms^42^, and *Bifidobacteria* agar (Himedia) for *Bifidobacteria*. Lactobacilli and coliforms were cultured anaerobically in an anaerobic jar on relevant agar plates for 24 h at 37 °C, while *Bifidobacteria* and Clostridia were incubated on the plate for 24 to 48 h. Each plate's bacterial colony was counted and expressed as log_10_ CFU/g of fresh feces. The ratio of lactobacilli to coliforms was determined by dividing the colony-forming units (CFU) of lactobacilli by the CFU of coliforms.

### Estimation of antioxidant status, humoral and cell-mediated immune response

Before morning feeding, 10 mL of blood was drawn from each calf's jugular vein at monthly interval in a clean, numbered vacutainer that contained acid-citrate dextrose. The vials were properly mixed with anticoagulant immediately after collection, preserved in an ice box, and brought to the lab for further analysis^33^. The hemolysate and RBC suspension were prepared according to the previously described methodology^43,44^ and stored at − 20 °C until the subsequent antioxidant assay by selected parameters.The catalase assay was performed using the spectrophotometric protocol of Aebi^45^. According to the Madesh and Balasubramanian technique^46^, after proper dilution, the activity of SOD in the samples of hemolysate was assessed using nitroblue tetrazolium as a substrate. Additionally, the glutathione peroxidase (GPx) activity was measured spectrophotometrically using Paglia and Valentine's method^47^.

Calves were injected intramuscularly with 1 mL of a 10% washed chicken RBC suspension (C-RBC) in 0.15 M NaCl as an antigen to assess their humoral immune (HI) response after 4 weeks (d 28) of the feeding trial. Serum samples were taken on days 0 (before injection), 7, 14, 21, and 28 to estimate the antibodies’ titer and stored at − 20 °C to perform antibody titer estimation using the hemagglutination (HA) previously reported by Wegmann and Smithies^48^. The HA titer measurement was performed after 3 h at room temperature and expressed as log_2_.

All animals were injected intradermally with phytohaemagglutinin-P (PHA-P) on the 58th day of the study to measure the thickness of the skin indurations and evaluate the cell-mediated immunity by the method suggested by Masucci et al.^49^. The skin area (upper side of the shoulder) to be tested was cleaned and shaved 24 h before performing the delayed-type hypersensitivity (DTH) test. On either side of the selected region, a black marker pen was used to ring a space of roughly one square cm. On one side of the region, each animal received an intradermal injection of 100 μL of phytohaemagglutinin-P [PHA-P (50 μg/100 μL PBS), Sigma, St. Louis, MO, USA] solution, while the opposite side received an injection of normal saline solution as a control. Following that, the thickness of the skin was measured at 6, 12, 18, and 24 h post-inoculation. A digital vernier caliper with a measurement range of 0–150 mm was used to determine the skin's thickness and depict the basal (0 h) value. The thickness was represented as an absolute (mm) increase over the pre-inoculation base value at 0 h.

### Statistical analysis

The Statistical Package for the Social Sciences (SPSS for Windows, v26.0; SPSS Inc., Chicago, IL, USA) was used to analyze all of the generated data. A one-way ANOVA was used to analyze data from the digestion experiment, dry matter intake, and body weight change. Morphometry parameters and immunological parameters (cell –mediated and humoral immunity) were analysed using a two-way ANOVA with repeated measure analysis. General linear model (GLM) approaches were used to assess the experimental data of parameters that were periodically collected (antioxidant concentration, fecal score, metabolites, microbiota, and short-chain fatty acids) with the fixed effects of treatments, time/period, and treatments × time/period. Pairwise comparisons of the mean values were tested by Tukey’s test at the significance level (*p* < 0.05). The mean and standard error mean of each set of data are shown for each parameter.

## Results and discussion

### Growth, structural growth measurements, and nutrient utilization

Data presented in Table 2 indicated that BW net gain (kg) was significantly (*p* < 0.05) higher in LEC (20.77 ± 0.36 kg), followed by AEC (19.46 ± 0.49 kg) and NEC (18.61 ± 0.27 kg), and the lowest value in CON (17.16 ± 0.19 kg). The average daily gain (ADG; g/d) followed a similar trend as reported in the net gain in BW (LEC > AEC > NEC > CON). Average DMI (g/d) was significantly higher (*p* < 0.05) in LEC than in CON, whereas NEC and AEC had intermediate values. In addition to this, feed efficiency (FE%) was significantly higher (*p* < 0.05) in LEC, followed by AEC, NEC, and CON. Supplementation had no effect (*p* > 0.05) on the apparent digestibility coefficient of several nutrients (DM, OM, CP, NDF, and ADF). However, a trend ( *p* = 0.098) was observed for the digestibility coefficient of EE, which was found to be 83.75% in LEC, 82.93% in AEC, 80.69% in NEC, and 79.14 in CON. The average initial values for different morphometry parameters (structural body measurements), viz., body length, initial heart girth, hip height, and wither height, were statistically similar, and the final figures noted at the end of the experiment were found significantly (*p* < 0.05) different (Table 3). The overall weekly averages of different structural body measurements, viz*.,* body length, heart girth, hip height, and wither height, in different groups indicating that all probiotic-treated groups had higher values as compared to control in response to probiotic feeding.

**Table 2:** Effect of supplementation of probiotics on growth performance and nutrient utilization in calves.

Attributes	Dietary groups				P values
	CON	NEC	AEC	LEC	
Initial BW (kg)	24.22 ± 0.95	24.25 ± 1.12	24.42 ± 0.93	24.40 ± 0.56	0.998
Final BW (kg)	41.38^a^ ± 0.99	42.86^ab^ ± 1.06	43.88^ab^ ± 0.86	45.17^b^ ± 0.43	< 0.05
Net gain (kg)	17.16^a^ ± 0.19	18.61^b^ ± 0.27	19.46^b^ ± 0.49	20.77^c^ ± 0.36	< 0.05
^1^ADG (g/d)	286.00^a^ ± 3.35	310.17^b^ ± 4.54	324.33^b^ ± 8.13	346.19^c^ ± 6.02	< 0.05
^2^ADMI(g/d)	715.84^a^ ± 6.28	749.15^b^ ± 3.31	766.85^b^ ± 3.42	770.28^b^ ± 5.52	< 0.05
^3^Feed efficiency (%)	39.95^a^ ± 0.53	41.40^b^ ± 1.37	42.29^bc^ ± 2.38	44.94^c^ ± 1.09	< 0.05
Apparent digestibility of nutrients (%)					
DM	68.08 ± 1.53	69.55 ± 1.95	71.75 ± 1.46	73.23 ± 1.13	0.128
OM	71.37 ± 3.06	72.69 ± 1.58	73.64 ± 1.45	75.80 ± 1.23	0.434
CP	68.88 ± 1.82	70.05 ± 2.31	71.98 ± 1.21	73.60 ± 1.14	0.180
EE	79.14 ± 1.56	80.69 ± 2.11	82.93 ± 1.89	83.75 ± 2.21	0.098
NDF	65.98 ± 1.74	67.56 ± 1.92	68.82 ± 1.34	71.01 ± 1.04	0.296
ADF	57.07 ± 2.04	58.37 ± 2.53	59.99 ± 3.21	62.31 ± 2.63	0.362

Values with different superscripts are significantly different from each other (*p* < 0.05). CON: basal diet without probiotics; NEC: non-encapsulated probiotics; AEC: air-dried encapsulated probiotics; LEC: lyophilized encapsulated probiotics. SEM (Standard error of mean).

^1^ADG = (kg of final BW − kg of initial BW)/experimental days (60 days).

^2^ADMI = (offered DM − residual DM)/experimental days (60 days).

^3^Feed efficiency = [Average daily gain (kg/day)/Dry matter intake (kg/ day)] × 100%. BW, body weight; ADG, average daily gain; ADMI, average dry matter intake; FCE, feed conversion efficiency; DM, dry matter; OM, organic matter; CP, crude protein; EE, ether extract; NDF, neutral detergent fibre; ADF, acid detergent fibre.

**Table 3:** Effect of probiotics supplementation on morphometry parameters in different groups of calves.

Attributes	CON	NEC	AEC	LEC	Period mean	T	D	T × D
Body length (cm)								
Initial	56.67 ± 1.05	57.00 ± 1.03	56.92 ± 0.88	57.08 ± 0.90	56.92^p^ ± 0.97	< 0.001	< 0.001	0.996
1st week	59.08 ± 0.45	60.50 ± 1.12	60.75 ± 1.26	61.00 ± 1.24	60.33^q^ ± 1.18			
2nd week	61.75 ± 0.89	63.75 ± 0.89	64.58 ± 1.02	64.92 ± 0.97	63.75^r^ ± 0.94			
3rd week	63.67 ± 0.91	66.58 ± 1.08	68.33 ± 2.45	68.50 ± 1.15	66.77^ s^ ± 1.40			
4th week	65.83 ± 0.90	69.33 ± 1.02	71.21 ± 2.14	71.33 ± 1.26	69.43^t^ ± 1.33			
5th week	69.00 ± 0.74	72.33 ± 1.12	73.83 ± 2.01	73.92 ± 1.37	72.27^u^ ± 1.31			
6th week	72.08 ± 0.81	75.00 ± 1.04	76.08 ± 2.05	76.67 ± 1.17	74.96^v^ ± 1.27			
7th week	75.95 ± 1.31	77.67 ± 1.01	78.50 ± 1.93	79.50 ± 0.99	77.90^w^ ± 1.31			
Final	77.94 ± 0.40	79.68 ± 0.89	81.27 ± 2.15	82.50 ± 1.00	80.35^x^ ± 1.35			
Average	66.87^a^ ± 0.70	69.09^b^ ± 1.02	70.16^b^ ± 1.77	70.60^b^ ± 1.12				
Wither height (cm)								
Initial	74.83 ± 0.76	75.08 ± 0.90	75.5 ± 1.29	75.67 ± 1.14	75.27^p^ ± 1.02	< 0.001	< 0.001	0.993
1st week	76.91 ± 0.34	78.83 ± 0.65	79.33 ± 0.60	79.58 ± 0.80	78.67^p^ ± 0.60			
2ndweek	79.00 ± 1.31	82.66 ± 0.57	82.83 ± 0.33	82.91 ± 1.02	81.85^q^ ± 0.81			
3rd week	81.08 ± 1.00	85.08 ± 0.57	85.17 ± 0.69	85.33 ± 0.85	84.17^r^ ± 0.78			
4th week	83.16 ± 0.72	87.50 ± 0.52	87.91 ± 0.52	88.08 ± 0.93	86.67^ s^ ± 0.67			
5th week	86.00 ± 0.87	89.75 ± 0.98	89.58 ± 0.93	90.33 ± 0.98	88.91^t^ ± 0.94			
6th week	89.08 ± 0.74	92.00 ± 1.01	92.33 ± 1.01	92.67 ± 1.17	91.52^u^ ± 0.98			
7th week	90.35 ± 0.64	94.16 ± 1.05	95.17 ± 1.26	95.42 ± 1.35	93.77^v^ ± 1.03			
Final	92.11 ± 0.57	95.77 ± 1.09	97.02 ± 1.28	97.47 ± 1.40	95.67^u^ ± 1.08			
Average	83.62^a^ ± 0.77	86.76^b^ ± 0.81	87.21^b^ ± 0.88	87.56^b^ ± 1.07				
Heart girth (cm)								
Initial	75.50 ± 0.85	75.92 ± 0.91	76.41 ± 1.08	75.41 ± 0.89	76.06^p^ ± 0.93	< 0.001	< 0.001	1.000
1st week	77.58 ± 0.34	77.54 ± 0.73	79.33 ± 0.44	79.58 ± 0.81	78.51^q^ ± 0.58			
2ndweek	78.00 ± 1.32	80.17 ± 0.60	80.50 ± 1.80	81.25 ± 0.96	79.98^q^ ± 1.17			
3rd week	80.00 ± 1.00	82.67 ± 0.67	83.16 ± 1.23	83.41 ± 1.03	82.31^r^ ± 0.98			
4th week	82.50 ± 1.06	85.08 ± 0.66	85.58 ± 1.16	85.67 ± 1.31	84.71^ s^ ± 1.05			
5th week	85.33 ± 0.92	87.50 ± 0.73	88.08 ± 1.00	88.50 ± 1.40	87.35^t^ ± 1.01			
6th week	89.08 ± 0.57	89.75 ± 0.73	90.75 ± 1.13	90.92 ± 1.04	90.13^u^ ± 0.87			
7th week	92.21 ± 0.65	92.50 ± 0.82	93.66 ± 1.17	93.00 ± 1.06	93.03^v^ ± 0.93			
Final	93.50 ± 0.40	94.25 ± 0.91	95.50 ± 1.28	95.90 ± 1.19	94.87^w^ ± 0.95			
Average	83.78^a^ ± 0.79	85.04^b^ ± 0.75	85.89^b^ ± 1.14	86.15^b^ ± 0.9				
Hip height (cm)								
Initial	73.08 ± 1.31	74.08 ± 1.11	74.58 ± 1.14	74.83 ± 1.19	74.15^p^ ± 1.19	< 0.001	< 0.001	0.999
1st week	74.83 ± 0.55	76.83 ± 1.31	77.41 ± 1.23	78.00 ± 1.07	76.77^q^ ± 1.04			
2nd week	76.66 ± 1.34	79.42 ± 1.16	81.25 ± 1.01	81.41 ± 1.45	79.69^r^ ± 1.24			
3rd week	78.83 ± 1.24	82.42 ± 1.16	84.25 ± 0.79	84.42 ± 1.50	82.50^ s^ ± 1.17			
4th week	81.00 ± 1.20	85.25 ± 1.06	86.08 ± 1.36	86.58 ± 1.54	84.73^t^ ± 1.29			
5th week	83.33 ± 1.11	88.25 ± 1.05	88.34 ± 1.14	88.67 ± 1.55	87.15^u^ ± 1.21			
6th week	86.16 ± 1.16	90.58 ± 1.23	90.91 ± 1.14	91.08 ± 1.53	89.68^v^ ± 1.27			
7th week	89.79 ± 1.08	92.92 ± 1.24	94.25 ± 1.01	93.70 ± 1.40	92.84^w^ ± 1.18			
Final	91.8 ± 0.58	95.40 ± 1.22	96.18 ± 1.11	97.81 ± 1.60	95.29^x^ ± 1.13			
Average	81.72^a^ ± 1.06	85.02^b^ ± 1.17	85.92^b^ ± 1.10	86.35^b^ ± 1.42				

^a,b^Values with different superscripts are significantly different from each other in the same row (p < 0.05). ^p–^^x^Values with different superscripts are significantly different from each other in the same column (p < 0.05). CON: basal diet without probiotics; NEC: non-encapsulated probiotics; AEC: air-dried encapsulated probiotics; LEC: lyophilized encapsulated probiotics; T: Treatment; D: Period; T*D: Treatment and Period interaction; *Structural growth measurements: average weekly values.

In the present study, improved growth performance of calves in the probiotics-supplemented group may be related to a higher DM intake, which in turn results in more nutrient supply that facilitates higher body growth. These beneficial microbes may harvest energy from the undigested feed that is resistant to indigenous microflora. The possible explanation may be due to the better colonizing capacity of host-specific lactobacilli strains, which favors the growth of beneficial symbionts and maintains gut epithelial membrane integrity. Moreover, probiotics also competitively exclude pathogenic bacteria before adhesion, thereby alleviating the physiological stress on the animal and making them more resilient to different stress factors encountered during their early life^50^. Similarly to this study, Sharma et al.^1^ evaluated the role of mannan-oligosaccharides (MOS) and *Lactobacillus acidophilus* feeding in calves and concluded that the treatment groups outperformed the control group in terms of body weight gain, nutrient intake, and feed conversion efficiency (*p* < 0.05). Recently, Ms et al.^51^ reported that supplementation ofencapsulated probiotics significantly increased average daily gain in treated animals as compared to the control young suckling calves**.** In consonance with the present findings, Rai et al.^52^ fed Jersey crossbred calves up to 3 months of age with fermentable synbiotics (*Lactobacillus rhamnosus* NCDC 298 and fructooligosaccharides) and found that probiotic and FOS-fed calves had increased average dry matter intake and growth performance of the calves compared to the control animals. Similar to this, Varada et al.^20^ carried out a study to look at the performance, immunity, and specific gut health indices of Murrah buffalo calves supplemented with autochthonous *Limosilactobacillus reuteri* BFE7 and *Ligilactobacillus salivarius* BF17 probiotic consortia. The study revealed that including probiotics in the diet for 60 days enhanced these parameters considerably *(p* < 0.05) when compared to the control group. However, contrary to these results, according to Zhang et al.^53^, there was no discernible change between dairy calves given *Lactobacillus plantarum* or *Bacillus subtilis* in terms of DMI or ADG (*p* < 0.05). Recently, Kumar et al.^9^ reported that pre-ruminant calves' ADG was unaffected by the administration of lyophilized, microencapsulated, or fermented milk prepared with *Lactobacillus acidophilus* NCDC15.

These discrepancies in inferences may be due to the type, processing, bacterial strains, dosage of probiotics, health status, rearing conditions, breed of animal, methods of probiotic feeding, etc. Higher dry matter intake and improved FCE offer more nutrients for skeletal deposition. Because the animals were in an active growth phase when the probiotics were provided, the findings were in the expected line. In consonance with the present study, Singh et al. ^13^ found that the addition of *Lactobacillus acidophilus* NCDC15 and *Lactobacillus reuteri* BFE7 with *Cichorium intybus* root powder significantly improved structural body measurements. Similarly, Sharma et al.^1^ reported that supplementation of mannan-oligosaccharides (MOS) and *Lactobacillus acidophilus* in calves increased structural growth parameters (like body length, hip height, heart girth, and wither height) (*p* < 0.05). On the contrary, Mokhber-Dezfouli et al.^54^ and Riddel et al.^55^ reported that probiotic supplementation did not affect body length, hip height, heart girth and wither height in dairy calves.

In this investigation, LEC outperformed then NEC; this might have occurred when probiotics were microencapsulated, thereby enhancing bacterial survival. Due to its insolubility in acidic pH, the microencapsulation material shields the probiotic live cells from gastric acid during their gastric transit; however, in the intestine, it releases the probiotics cells because of its swelling property in alkaline pH^56,57^. Nevertheless, the probiotic that has not been encapsulated in gastric juice loses its viable state before it reaches at intended destination. Furthermore, it has been noted that the prebiotic substances added during the microencapsulation process provide additional energy and a carbon source for the probiotic bacteria to thrive and proliferate in the gut^57^.On the other hand, LEC was better than AEC may be due to freeze drying being more effective in maintaining microbial viability and moisture level^27^.

### Fecal characteristics

#### Fecal consistency index, moisture, and pH

**Figure 1:**
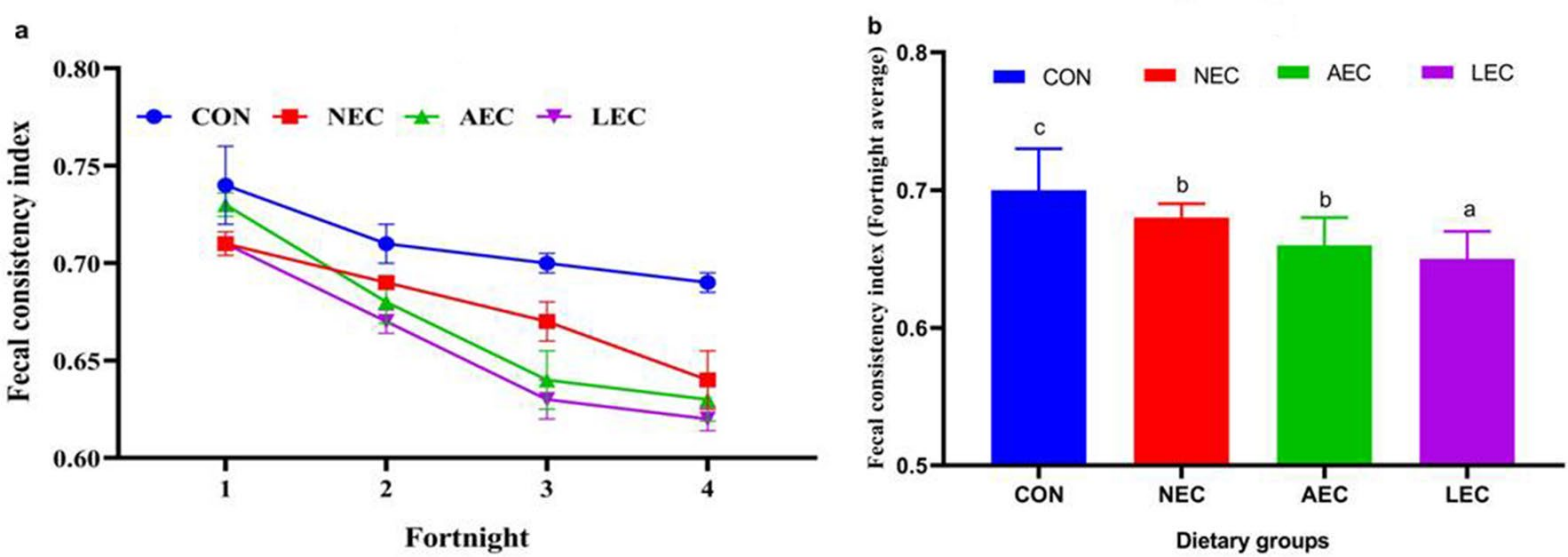
Effect of supplementation of probiotics on faecal consistency index during different fortnight (**a**) and average faecal consistency index (**b**) in calves. Values with different superscripts are significantly different from each other (*p* < 0.05). CON: basal diet without probiotics; NEC: non-encapsulated probiotics; AEC: air-dried encapsulated probiotics; LEC: lyophilized encapsulated probiotics.

**Figure 2:**
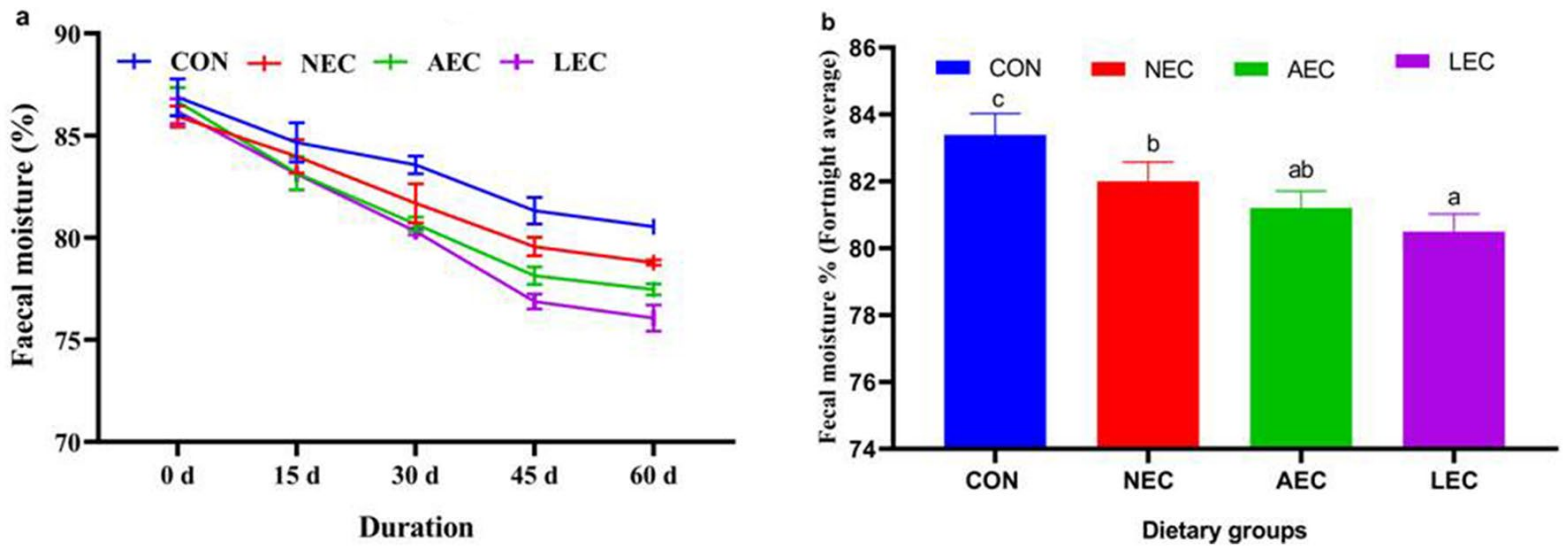
Effect of supplementation of probiotics on faecal moisture during different fortnight (**a**) and average faecal moisture (**b**) in calves. CON: basal diet without probiotics; NEC: non-encapsulated probiotics; AEC: air-dried encapsulated probiotics; LEC: lyophilized encapsulated probiotics.

**Figure 3:**
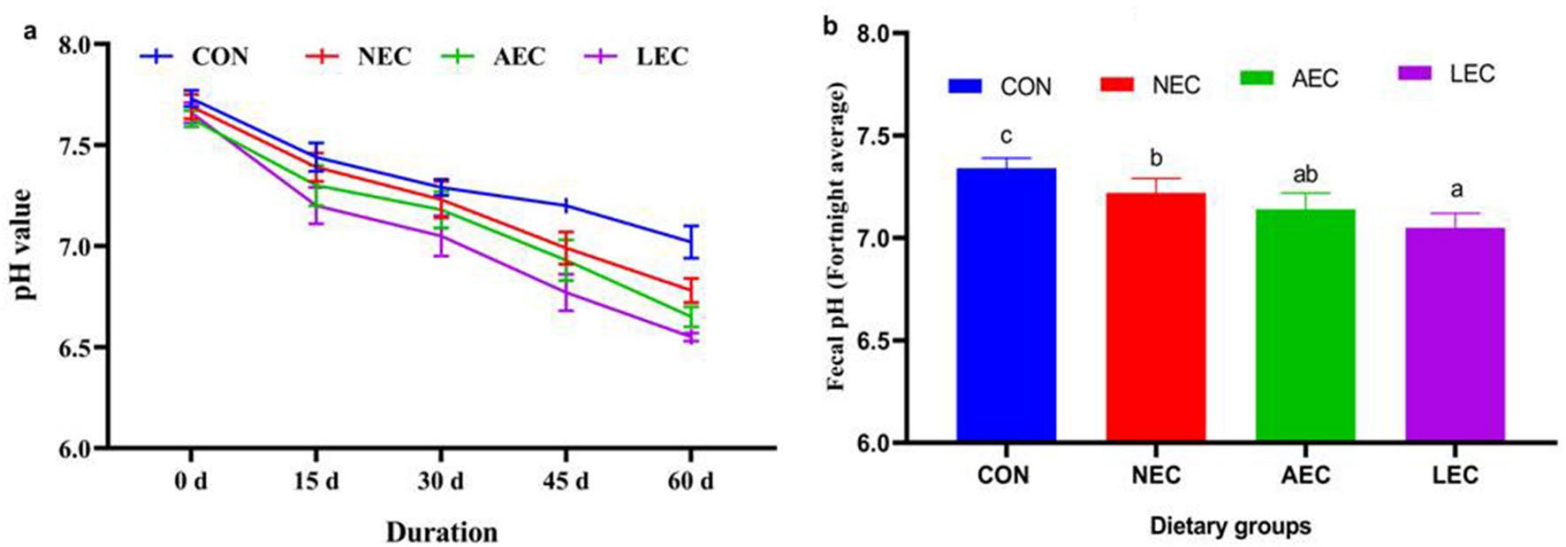
Effect of supplementation of probiotics on faecal pH during different fortnight (**a**) and average faecal pH (**b**) in calves. CON: basal diet without probiotics; NEC: non-encapsulated probiotics; AEC: air-dried encapsulated probiotics; LEC: lyophilized encapsulated probiotics.

The average fecal consistency index (FCI) of the different fortnights is depicted in Fig. 1a. The average FCI levels were comparable during the first fortnight and began to decline from that point in all the probiotic-supplemented groups. The average FCI values of all four fortnights (Fig. 1b) were lowest in the LEC group (0.65 ± 0.02) and then followed by AEC (0.66 ± 0.02), and NEC (0.68 ± 0.01), with the lowest values in control (0.70 ± 0.03). A similar trend was reported in the case of fecal moisture (%), as shown in Fig. 2a and Fig. 2b. The average fecal moisture (%) was decreased in the LEC group (80.50 ± 0.52), AEC (81.21 ± 0.50), and NEC (82.00 ± 0.58) than control (83.39 ± 0.64). The average values of fecal pH at different fortnights also differed upon period-wise comparison (Fig. 3a). Similarly, average fortnight fecal pH (Fig. 3b) was significantly (*p* < 0.05) reduced in LEC (7.05 ± 0.07), followed by NEC (7.22 ± 0.08) as compared to the control (7.34 ± 0.05), while values in the AEC group (7.14 ± 0.07) were comparable to NEC and LEC.

The feces consistency index is used to assess the frequency, regularity, and severity of diarrhea in calves. The decrease in feces consistency index in the treatment groups may be attributable to an increase in the colonization of lactic acid bacteria in the gut, which keeps the tight connection in place and prevents the adhesion of coliforms. The extra lactate produced in supplement groups by lactic acid bacteria colonization may be responsible for a reduction in fecal pH in probiotic-supplemented groups. In consonance with the present outcome, Bayatkoushar et al.^58^ reported that supplementation of lactic acid bacteria reduced fecal score, and pH as compared to the control group. Similarly, Lee et al.^59^ and Varada et al.^21^ revealed that probiotic supplementation substantially shortened (*p* < 0.05) the duration of diarrhea and the fecal score in treated groups compared to the control group. Sahu et al.^60^ reported that the administration of probiotics and prebiotics significantly (*p* < 0.05) improved fecal scores in Jersey crossbred calves.

#### Fecal metabolites and short-chain fatty acids

A perusal of the data tabulated in Table 4 on the fecal metabolites (ammonia and lactate) showed that supplementation of probiotics in different treatment groups was accompanied by a reduction (*p* < 0.05) in ammonia concentrations. The lowest average fecal ammonia concentration (µmol/g of fresh feces) was reported in LEC < AEC < NEC and the highest value in CON. Moreover, treatment and period interactions were also statistically different. An opposite picture was evident in the case of fecal lactate concentration. The LEC and NEC had a significantly (*p* < 0.05) increased average fecal lactate content as compared to the control, with AEC showing intermediate values of LEC and NEC (*p* < 0.05). Data regarding fecal short- chain fatty acids (SCFAs) given in Table 4 indicated that the average acetate concentrations (µmol/g of fresh feces) in the LEC, AEC, and NEC groups were significantly (*p* < 0.05) enhanced after feeding probiotics for 60d as compared to the control. The average propionate concentrations in the LEC, AEC, and NEC groups were also significantly (*p* < 0.05) higher than CON. The fecal butyrate concentration showed no significant difference (*p* > 0.05) among the different groups as a result of probiotic feeding.

**Table 4:** Effect of probiotics supplements on fecal metabolites, and short-chain fatty in calves.

Attributes	CON	NEC	AEC	LEC	Period mean	T	P	T × P
Ammonia (µmol/g of fresh faeces)								
0 d	5.68 ± 0.14	5.82 ± 0.20	5.64 ± 0.15	5.68 ± 0.13	5.70^ s^ ± 0.16	< 0.001	< 0.001	< 0.001
15 d	5.63 ± 0.16	5.54 ± 0.12	5.50 ± 0.13	5.41 ± 0.23	5.52^r^ ± 0.16			
30 d	5.61 ± 0.14	5.02 ± 0.04	4.88 ± 0.02	5.05 ± 0.02	5.17^q^ ± 0.06			
45 d	5.33 ± 0.10	4.88 ± 0.02	4.66 ± 0.03	4.66 ± 0.09	4.85^p^ ± 0.06			
60 d	5.22 ± 0.03	4.12 ± 0.14	3.80 ± 0.21	3.09 ± 0.26	4.06^p^ ± 0.16			
Average	5.50^c^ ± 0.12	5.08^b^ ± 0.10	4.90^ab^ ± 0.11	4.78^a^ ± 0.15				
Lactate (µmol/g of fresh faeces)								
0 d	2.99 ± 0.14	2.91 ± 0.10	3.06 ± 0.20	3.11 ± 0.06	3.02^p^ ± 0.13	< 0.001	< 0.001	0.573
15 d	3.14 ± 0.11	3.26 ± 0.11	3.31 ± 0.20	3.36 ± 0.07	3.27^q^ ± 0.12			
30 d	3.32 ± 0.05	3.49 ± 0.11	3.67 ± 0.18	3.74 ± 0.13	3.54^r^ ± 0.12			
45 d	3.29 ± 0.16	3.72 ± 0.09	3.87 ± 0.16	3.99 ± 0.09	3.75 ± 0.13			
60 d	3.49 ± 0.11	3.87 ± 0.11	3.99 ± 0.15	4.22 ± 0.06	3.89^ s^ ± 0.11			
Average	3.25^a^ ± 0.11	3.45^b^ ± 0.10	3.58^bc^ ± 0.18	3.69^c^ ± 0.08				
Acetate (µmol/g of fresh faeces)								
0 d	26.38 ± 0.82	29.09 ± 029	27.33 ± 0.45	28.42 ± 0.52	27.81^p^ ± 0.52	< 0.001	< 0.001	0.525
15 d	27.69 ± 1.00	29.77 ± 0.32	30.00 ± 0.16	30.10 ± 0.28	29.39^q^ ± 0.44			
30 d	28.62 ± 0.81	29.90 ± 0.65	30.20 ± 0.29	30.42 ± 1.04	29.79^q^ ± 0.70			
45 d	29.55 ± 0.72	30.01 ± 0.59	30.66 ± 0.56	31.30 ± 0.82	30.378^q^ ± 0.67			
60 d	30.48 ± 0.88	30.91 ± 0.44	32.86 ± 0.74	33.05 ± 0.59	31.82^r^ ± 0.66			
Average	28.54^a^ ± 0.84	29.93^b^ ± 0.46	30.21^b^ ± 0.44	30.66^b^ ± 0.65				
Propionate (µmol/g of fresh faeces)								
0 d	8.88 ± 0.28	9.33 ± 0.27	8.90 ± 0.22	9.41 ± 0.25	9.13^p^ ± 0.26	0.003	< 0.001	0.933
15 d	9.55 ± 0.24	9.91 ± 0.29	9.92 ± 0.28	9.85 ± 0.28	9.81^q^ ± 0.27			
30 d	10.26 ± 0.30	10.74 ± 0.39	10.95 ± 0.33	10.95 ± 0.29	10.73^r^ ± 0.33			
45 d	10.96 ± 0.26	11.36 ± 0.24	11.62 ± 0.29	11.72 ± 0.35	11.42^ s^ ± 0.28			
60 d	11.63 ± 0.26	12.12 ± 0.30	12.49 ± 0.30	12.83 ± 0.35	12.27^t^ ± 0.30			
Average	10.26^a^ ± 0.27	10.69^b^ ± 0.30	10.78^b^ ± 0.28	10.95^b^ ± 0.30				
Butyrate (µmol/g of fresh faeces)								
0 d	4.76 ± 0.28	4.45 ± 0.22	4.62 ± 0.24	4.79 ± 0.27	4.66^p^ ± 0.25	0.052	< 0.001	0.997
15 d	4.84 ± 0.23	4.87 ± 0.23	5.04 ± 0.20	5.22 ± 0.22	4.99^p^ ± 0.22			
30 d	5.48 ± 0.24	5.54 ± 0.28	5.73 ± 0.21	5.86 ± 0.20	5.65^q^ ± 0.23			
45 d	6.07 ± 0.15	6.19 ± 0.30	6.41 ± 0.24	6.54 ± 0.29	6.30^r^ ± 0.25			
60 d	6.70 ± 0.10	6.90 ± 0.26	7.21 ± 0.24	7.32 ± 0.24	7.03^ s^ ± 0.21			
Average	5.57 ± 0.20	5.59 ± 0.26	5.80 ± 0.23	5.95 ± 0.24				

^a,b^Values with different superscripts are significantly different from each other in the same row (*p* < 0.05). ^p–t^Values with different superscripts are significantly different from each other in the same column (*p* < 0.05). CON: basal diet without probiotics; NEC: non-encapsulated probiotics; AEC: air-dried encapsulated probiotics; LEC: lyophilized encapsulated probiotics; T: Treatment; D: Period; T*D: Treatment and Period interaction.

Supplementing with probiotics decreased fecal ammonia and increased fecal lactate. Animals produce ammonia during the colonic fermentation of protein, which is thought to occur at trace levels when probiotic bacteria are present. Additionally, when there is a shortage of energy sources, gut microorganisms convert amino acids into SCFA and ammonia to generate energy. Probiotic bacteria accelerate the digestion of carbohydrates that are unfriendly to native bacteria, which increases lactate synthesis^61^. On the other hand, lactic acid bacteria directly use glucose as a carbon source to make pyruvate through glycolysis during pure lactic acid fermentation. Lactate dehydrogenase then breaks down the pyruvate to produce lactic acid^62^. In consonance with the present findings, *L. acidophilus* fed group had significantly lower fecal ammonia levels and higher lactate levels than the control group^1^. Similarly, Singh et al.^13^ recently demonstrated that probiotic treatment decreased fecal ammonia levels while concurrently increasing the stool lactate levels as compared to the control. Khare et al.^63^ showed that the supplementation of prebiotics (chicory root powder) reduced the fecal ammonia and enhanced the fecal lactate in neonatal *Murrah* buffalo calves. On contrary to this, Qadis et al.^64^ observed no impact on lactate production following the supplementation of probiotics to Holstein’s calves.

The present study's improvement in SCFA levels is attributed to probiotics' improved colonization and adaptation in the calf gut. Because probiotics convert dietary fibers into SCFA, which is used to fuel colonocytes, liver cells, and peripheral tissues. The SCFA meets 10 to 30 percent energy requirement for maintaining the metabolic needs of the host animal^1^. These metabolites are also known to enhance gut integrity, lipid homeostasis, regulate glucose, and improve immune function. Our results concurred with the findings reported by Sharma et al.^1^, who found that *L. acidophilus* supplementation raised SCFA levels in *Murrah* buffalo calves. Similarly, Ohya et al.^65^ observed that supplementation with a freeze-dried probiotic product including *L. gallinarum* LCB12 and *Streptococcus bovis* LCB6 resulted in a considerable rise in levels of the fecal SCFAs, particularly acetic acid. On the contrary to this, Ramaswami et al.^66^ reported no change in the molar proportion of acetate, and propionate, while butyrate was significantly improved in *L. acidophilus* supplementation in the cross-bred calves.

### Select fecal microbiota and lactobacillus coliform ratio

**Figure 4:**
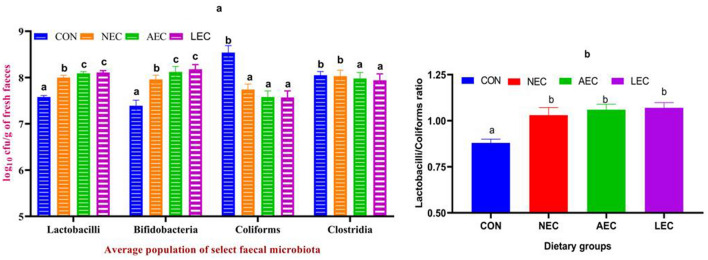
Effect of supplementation of probiotics on average microbiota population (**a**) and lactobacilli coliforms ratio (**b**) in calves. Values with different superscripts are significantly different from each other (*p* < 0.05). CON: basal diet without probiotics; NEC: non-encapsulated probiotics; AEC: air-dried encapsulated probiotics; LEC: lyophilized encapsulated probiotics.

There were no significant differences (*p* > 0.05) observed for the select fecal microbiota population of *Lactobacillus, Bifidobacterium, Coliform, and Clostridium* spp. at the beginning of the experiment*.* Feeding of probiotics in different groups induces a change in the populations of these microbiota (Supplementary Table 1). The average of five periodic collections (0, 15, 30, 45, and 60 d) was presented in Fig. 4a. The average population of *Lactobacilli* was significantly increased (*p* < 0.05) in all the treatment groups, and the enhancement was highest in LEC (8.11 ± 0.04) and AEC (8.09 ± 0.04), followed by NEC (8.00 ± 0.05), with the lowest value in control (7.58 ± 0.03). Additionally, a between-period comparison showed that the supplementation may have contributed to the greater *Lactobacillu*s count shown on days 15, 30, 45, and 60 as compared to control (Supplementary Table 1). Average fecal *Bifidobacterium* count followed a similar pattern, with the average values for CON, NEC, AEC, and LEC being 7.39 ± 0.12, 7.96 ± 0.09, 8.12 ± 0.12, and 8.18 ± 0.10, respectively. The average *Bifidobacterium* count was higher in LEC and AEC, followed by NEC with the lowest value in control. The present findings also indicated that *Bifidobacterial* count responded favorably to the *Lactobacilli* supplementation. The probiotic supplementation decreased the average fecal coliform count in NEC (7.74 ± 0.12), AEC (7.58 ± 0.13), and LEC (7.57 ± 0.14) as compared to the control group (8.54 ± 0.15). The period had a significant impact on the coliform population as well; it was lower on day 60 than the day 0 value. Similarly, when compared to the control group (8.05 ± 0.08), the average fecal *Clostridial* count was decreased in AEC (7.98 ± 0.13), and LEC (7.94 ± 0.14). The period had a significant impact on the *Clostridial* population; it was lower on day 60 than the day 0 value. The Lactobacilli and coliforms ratio (Fig. 4b) was also found to be statistically significant, with values < 1 in CON and values > 1 in all the probiotic-fed groups.

Due to their involvement in numerous immunological, nutritional, and physiological processes, the nature of the gut microbiota and its metabolites are essential for promoting gut health^67^. The current study demonstrates the predominance of beneficial bacteria and decreases the number of pathobionts in all the supplemented groups, with greater effects in the lyophilized and microencapsulated group as compared to the nonencapsulated probiotic group. The positive impacts in treatment groups may be because of competition for nutrients and inhibition of attachment by exogenous pathogenic microorganisms competing for binding sites in the intestine with beneficial bacteria. Moreover, it also creates an unfavorable environment (reduced pH, SCFA, antimicrobial peptides like bacteriocin, reuterin, hydrogen peroxide, etc.) in the gut, which curbs the pathobionts proliferation. The result of the present experiment was similar to Kumar et al.^9^, who reported that the supplementation of lyophilized and microencapsulated probiotics enhanced the health-promoting bacteria with a corresponding decrease in the pathobionts in calves. Similarly, Quezada-Mendoza et al.^68^ also reported that the feeding *Lactobacillus* and *Propionibacterium* spp. significantly increased the *Lactobacilli* counts in calves. *Lactobacillus* and *Bifidobacterium* are regarded as probiotics. The bifidobacterium strains in the intestinal lumen directly suppress the pathogens by generating lactic acid, H_2_O_2_, bacteriocins, and other inhibitory agents and by lowering nutrition competition for *Lactobacillus*. For this reason, *Bifidobacterium* reacted favourably to *Lactobacillus*^69^.

Dar et al.^70^ observed that supplementation of probiotics, prebiotics, and synbiotics significantly reduced the *E. coli* count in feces. *Lactobacillus* counts in feces are generally greater than coliform counts in healthy calves (LAB: coliforms ratio > 1); however, this relationship can vary substantially in calves suffering from diarrhoea^71^. The positive effects of LAB are most likely attributable to their growth in the intestinal system, which establishes microbiological protection, preventing the growth of harmful microbes. He et al.^72^ found, in contrast to these findings, that supplementing with yeast probiotics did not affect the feces bacterial community in Holstein's calves.

### Antioxidant indices

Antioxidant indices presented in Table 5 indicated that initially, superoxide dismutase activity was similar in all the animals. Feeding of probiotics induces an enhancement (*p* < 0.05) in SOD activity. The NEC, AEC, and LEC had significantly (*p* < 0.05) higher SOD activity than the control. The period-wise comparison also revealed the highest value at 60 d, followed by 30 d, with the lowest value at 0 d. Catalase (CAT) activity among groups was not affected by the probiotic supplementation in all the animals, however, a period-wise comparison showed that the levels were significantly greater (*p* < 0.05) at 60 d as compared to 30 d and 0 d. The activity of glutathione peroxidase (GPx) also remained comparable (*p* > 0.05) among the four groups, however, a period-wise comparison showed that the levels were significantly greater (*p* < 0.05) on day 60, followed by 30 d and 0 d.

**Table 5:** Effect of dietary supplementation of probiotics on serum antioxidant concentration in calves.

Attributes	Dietary groups				Mean	*P* values		
	CON	NEC	AEC	LEC		T	D	T*D
Super oxide dismutase (U/ml)								
0 d	5.33 ± 0.32	5.31 ± 0.39	5.30 ± 0.16	5.35 ± 0.27	5.32^p^ ± 0.28	< 0.001	< 0.001	0.011
30 d	5.52 ± 0.24	6.44 ± 0.19	6.65 ± 0.12	6.61 ± 0.10	6.30^q^ ± 0.16			
60 d	5.94 ± 0.31	7.32 ± 0.05	7.54 ± 0.10	7.57 ± 0.08	7.09^r^ ± 0.14			
Mean	5.60^a^ ± 0.29	6.35^b^ ± 0.21	6.50^b^ ± 0.13	6.51^b^ ± 0.15				
Catalase (nmol/min/ml)								
0 d	20.00 ± 0.40	19.72 ± 0.37	20.77 ± 0.49	20.90 ± 0.46	20.35^p^ ± 0.43	0.422	0.002	0.766
30 d	20.50 ± 0.61	20.69 ± 0.48	21.00 ± 0.43	20.37 ± 0.25	20.64^p^ ± 0.44			
60 d	21.07 ± 0.80	22.03 ± 0.50	21.88 ± 0.50	21.99 ± 0.91	21.74^p^ ± 0.68			
Mean	20.52 ± 0.60	20.81 ± 0.45	21.22 ± 0.47	21.09 ± 0.54				
Glutathione peroxidase (nmol/min/ml)								
0 d	4.87 ± 0.25	4.92 ± 0.12	4.95 ± 0.13	5.03 ± 0.15	4.94^p^ ± 0.16	0.855	0.021	1.000
30 d	5.18 ± 0.34	5.21 ± 0.26	5.24 ± 0.11	5.26 ± 0.12	5.22^q^ ± 0.21			
60 d	5.21 ± 0.05	5.23 ± 0.11	5.28 ± 0.07	5.32 ± 0.06	5.26^q^ ± 0.08			
Mean	5.09 ± 0.21	5.12 ± 0.16	5.15 ± 0.10	5.20 ± 0.11				

Means bearing different superscripts in a row (a,b,c) or coloum (p,q,r) within interaction differ significantly.

CON: basal diet without probiotics; NEC: non-encapsulated probiotics; AEC: air-dried encapsulated probiotics; LEC: lyophilized encapsulated probiotics.

The body typically maintains a balance between oxidants (generated during typical metabolic processes like reactive oxygen and nitrogen species) and antioxidants (like peroxidase, endogenous superoxide dismutase, reduced glutathione, and catalase). Reactive oxygen species (ROS) are produced by regular body systems during respiration in aerobic species due to stress or other factors such as weaning, age, and high temperature, which may oxidize host macromolecules like proteins, mutate DNA, oxidize membrane phospholipids, and alter low-density lipoproteins that cause an imbalance of the antioxidant status of the host. Animal health suffers as a result of elevated ROS. Probiotics in the diet have been demonstrated to significantly reduce the negative effects of oxidative stress by increasing the activity of antioxidant enzymes. Probiotics' antioxidant qualities primarily include chelating metal ions, scavenging free radicals, regulating the production of antioxidant enzymes, and influencing gut flora. Probiotics have the ability to influence signalling pathways, including NF-κB, MAPK, Nrf-2, and SIRTs, at the molecular level, hence producing antioxidant benefits^73,74^. Similar to the current investigation, Ojha et al.^15^ found that supplementation with *Lactobacillus acidophilus* improved superoxide dismutase (SOD) activity, while maintaining catalase and glutathione peroxidase (GPx) activity in all groups. Likewise, Wang et al.^75^ reported that feeding microencapsulated probiotics and prebiotics significantly increased the total antioxidant capacity in the broiler chickens.

### Immune response

**Figure 5:**
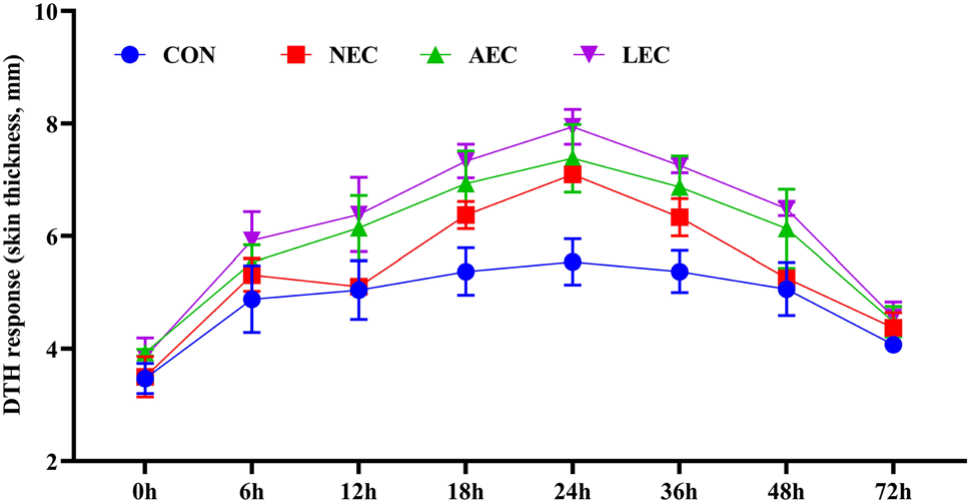
Effect of supplementation of probiotics on delayed type hypersensitivity (DTH) response to intradermal phytohaemaglutinin-P(PHA-P) in calves (significance: T < 0.001; P < 0.001; T*P = 0.774). CON: basal diet without probiotics; NEC: non-encapsulated probiotics; AEC: air-dried encapsulated probiotics; LEC: lyophilized encapsulated probiotics.

The findings concerning the CMI in terms of delayed non-specific hypersensitive reaction to phytohaemagglutinin-P are shown in Fig. 5. The results indicated that all the animals showed a positive DTH response to PHA-P. The value of skin induration peaked at 24 h after the inoculation and then progressively decreased until 72 h later. Average absolute skinfold thickness increased significantly (*p* < 0.05) in the probiotic-fed groups than in the control group (LEC > AEC > NEC > CON). The average absolute values (mm) were 4.85 ± 0.39, 5.41 ± 0.23, 5.92 ± 0.46, and 6.22 ± 0.33 for the CON, NEC, AEC, and LEC groups, respectively.

**Figure 6:**
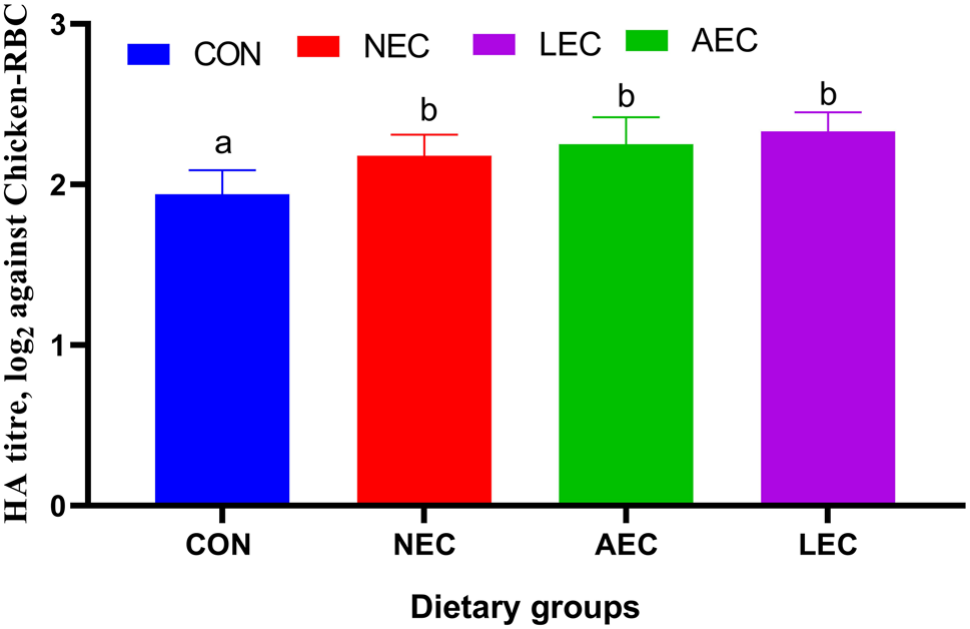
Effect of supplementation of probiotics on humoral immune response (antibody titer against Chicken- RBC) in calves (significance: T < 0.003; P < 0.001; T*P = 0.113). CON: basal diet without probiotics; NEC: non-encapsulated probiotics; AEC: air-dried encapsulated probiotics; LEC: lyophilized encapsulated probiotics.

The HIR was identified using the HA test as the antibody response to the antigen (chicken-RBC). The antibody response gradually increased up to 21 d after the inoculation of Chicken-RBC in all the groups (data not shown). The average values of HIR (HA titer, log_2_ against chicken-RBC) as displayed in Fig. 6 were 1.94 ± 0.15, 2.18 ± 0.13, 2.25 ± 0.17, and 2.33 ± 0.12 in CON, NEC, AEC, and LEC groups, respectively, which clearly indicated that antibody response against the chicken-RBC was significantly (*p* < 0.05) higher in all the probiotics-fed groups than the control.

Due to a number of both particular and general defense mechanisms that prevent pathogens from invading the intestinal mucosa, the GIT coexists with the local microbiota. Because > 70% of immune cells are found in the GIT, numerous studies have already been done that demonstrate the importance of gut the microbiota in immunological homeostasis and immune-associated disease states. The rise in skin fold thickness in the treatment groups suggested an improvement in cellular immunity, which may have been brought about by increased T lymphocyte activation and proliferation or by the secretion of cytokines that regulate several cellular immunity pathways. The results of this investigation were supported by Dar et al.^70^, who demonstrated that supplementing probiotics, prebiotics, and synbiotics resulted in better cell-mediated immunity. In contrary to the present findings, Roodposhti and Dabiri^76^, examined that the cell-mediated response did not differ significantly across the different groups of calves treated with probiotics. The B lymphocyte lineage cells' released antibodies, generated in response to an antigen (chicken-RBC), are the immune factor that mediates the HI response. Mohamadi and Dabiri^77^, observed that the HI response increased in the supplemented calves receiving the injection of ovalbumin on the 56th day as compared to the control. On the contrary, Ms et al.^51^ reported IgG concentrations had no significant difference between encapsulated and non-encapsulated probiotics on pre-ruminant calves.

## Conclusions

The results of the current study demonstrated that probiotic *Limosilactobacillus reuteri* SW23 supplementation offered neonatal calves with health benefits in various ways, including improved immune response in probiotic-fed groups, which ultimately resulted in better performance and improved gut health of newborn calves, leading to a lower incidence of diarrhea. The results also showed that among all supplemented groups, calves in the lyophilized microencapsulated group outperformed air-dried microencapsulated and non-microencapsulated groups in terms of average daily gain, dry matter intake, and gut health indicators.

### Supplementary Information

Supplementary Table S1.

## Data Availability

All the data generated during the experiment are given in the manuscript.
