# Supplementary material for: Non-encapsulated, encapsulated, and lyophilized probiotic Limosilactobacillus reuteri SW23 influenced the growth and gut health in calves
Source: Sci Rep. 2024 Apr 1;14:7657. doi: 10.1038/s41598-024-57353-y (PMC10984972; doi:10.1038/s41598-024-57353-y)
Supplement: Supplementary file 1 — Supplementary Table S1. [file 41598_2024_57353_MOESM1_ESM.docx]

**Journal name: Scientific reports**

**Title: Non-encapsulated, encapsulated, and lyophilized probiotic *Limosilactobacillus reuteri* SW23 influenced the growth and gut health in calves**

Manish Yadav, Sachin Kumar*, Yash Parsana, Nutan Chauhan, Nitin Tyagi, Goutam Mondal and Ashis Kumar Samanta

Rumen Biotechnology Laboratory

Division of Animal Nutrition

ICAR-National Dairy Research Institute, Karnal-132001, Haryana, INDIA

***Corresponding author**

Email: arensachin@gmail.com (Sachin Kumar); Contact number: +91-1842259069

**Table S1 Effect of dietary supplementation of probiotics on faecal bacteria enumeration (log10CFU/g) in indigenous cattle calves**

| **Attributes** | **Dietary groups** | | | | **Mean** | ***P* values** | | |
| --- | --- | --- | --- | --- | --- | --- | --- | --- |
|  | **CON** | **NEC** | **AEC** | **LEC** |  | **T** | **D** | **T × D** |
| ***Lactobacillu*s (log_10_ CFU/g of fresh faeces)** | | | | | | | | |
| **0 d** | 7.89±0.03 | 7.81±0.05 | 7.90±0.04 | 7.82±0.03 | 7.85^p^±0.04 | < 0.001 | < 0.001 | < 0.001 |
| **15 d** | 7.75±0.05 | 7.85±0.04 | 7.96±0.05 | 7.98±0.05 | 7.88^pq^±0.05 |  |  |  |
| **30 d** | 7.54±0.03 | 8.00±0.06 | 8.06±0.04 | 8.16±0.05 | 7.94^qr^±0.05 |  |  |  |
| **45 d** | 7.48±0.01 | 8.13±0.05 | 8.25±0.02 | 8.26±0.05 | 8.03^rs^±0.03 |  |  |  |
| **60 d** | 7.22±0.04 | 8.23±0.04 | 8.28±0.04 | 8.31±0.04 | 8.01^s^±0.04 |  |  |  |
| **Average** | 7.58^a^±0.03 | 8.00^b^±0.05 | 8.09^c^±0.04 | 8.11^c^±0.04 |  |  |  |  |
| ***Bifidobacterium* (log_10_ CFU/g of fresh faeces)** | | | | | | | | |
| **0 d** | 7.88±0.14 | 7.75±0.04 | 7.87±0.13 | 7.88±0.14 | 7.85^p^±0.11 | < 0.001 | 0.002 | < 0.001 |
| **15 d** | 7.76±0.08 | 7.85±0.08 | 7.97±0.08 | 7.99±0.11 | 7.89^p^±0.09 |  |  |  |
| **30 d** | 7.65±0.10 | 7.99±0.13 | 8.13±0.15 | 8.23±0.09 | 8.00^q^±0.12 |  |  |  |
| **45 d** | 7.28±0.09 | 8.05±0.09 | 8.31±0.12 | 8.35±0.12 | 7.99^q^±0.11 |  |  |  |
| **60 d** | 6.31±0.17 | 8.16±0.12 | 8.29±0.10 | 8.45±0.05 | 7.80^p^±0.11 |  |  |  |
| **Average** | 7.39^a^±0.12 | 7.96^b^±0.09 | 8.12^c^±0.12 | 8.18^c^±0.10 |  |  |  |  |

| ***Coliform* (log10 CFU/g of fresh faeces)** | | | | | | | | | | | | | |
| --- | --- | --- | --- | --- | --- | --- | --- | --- | --- | --- | --- | --- | --- |
| **Attributes** | | **CON** | | **NEC** | | **AEC** | | **LEC** | | **Period mean** | **T** | **D** | **T × D** |
| **0 d** | | 8.12±0.10 | | 8.10±0.13 | | 8.15±0.10 | | 8.14±0.09 | | 8.13^r^±0.11 | < 0.001 | < 0.001 | < 0.001 |
| **15 d** | | 8.56±0.23 | | 7.98±0.16 | | 7.88±0.18 | | 7.88±0.12 | | 8.07^r^±0.17 |  |  |  |
| **30 d** | | 8.88±0.17 | | 7.86±0.07 | | 7.71±0.16 | | 7.72±0.05 | | 8.04^r^±0.13 |  |  |  |
| **45 d** | | 8.85±0.06 | | 7.52±0.14 | | 7.23±0.08 | | 7.15±0.19 | | 7.69^q^±0.12 |  |  |  |
| **60 d** | | 8.34±0.16 | | 7.22±0.08 | | 6.92±0.13 | | 7.01±0.25 | | 7.37^p^±0.16 |  |  |  |
| **Average** | | 8.54 ^b^±0.15 | | 7.74 ^a^±0.12 | | 7.58 ^a^±0.13 | | 7.57 ^a^±0.14 | |  |  |  |  |
| ***Clostridium* (log10 CFU/g of fresh faeces)** | | | | | | | | | | | | | |
| **0 d** | 8.17±0.03 | | 8.19±0.07 | | 8.17±0.08 | | 8.15±0.07 | | 8.17^t^±0.07 | | < 0.001 | < 0.001 | 0.040 |
| **15 d** | 8.14±0.10 | | 8.11±0.16 | | 8.11±0.07 | | 8.03±0.02 | | 8.10^s^±0.09 | |  |  |  |
| **30 d** | 8.07±0.04 | | 8.02±0.02 | | 8.01±0.14 | | 7.96±0.23 | | 8.02^r^±0.11 | |  |  |  |
| **45 d** | 7.97±0.10 | | 7.95±0.23 | | 7.89±0.16 | | 7.86±0.24 | | 7.92^q^±0.18 | |  |  |  |
| **60 d** | 7.91±0.12 | | 7.84±0.15 | | 7.72±0.19 | | 7.71±0.17 | | 7.79^p^±0.16 | |  |  |  |
| **Average** | 8.05^b^±0.08 | | 8.03^b^±0.13 | | 7.98^a^±0.13 | | 7.94^a^±0.14 | |  | |  |  |  |

Means bearing different superscripts in a row (a,b,c) or coloum (p,q,r,s,t) within interaction differ significantly. CON: basal diet without probiotics; NEC: non-encapsulated probiotics; AEC: air-dried encapsulated probiotics; LEC: lyophilized encapsulated probiotics; T: Treatment; D: Period; T*D: Treatment and Period interaction
